# Coronary Computed Tomography Angiography: Beyond Obstructive Coronary Artery Disease

**DOI:** 10.3390/life13051086

**Published:** 2023-04-26

**Authors:** Elisabetta Tonet, Alberto Boccadoro, Marco Micillo, Marta Cocco, Alberto Cossu, Graziella Pompei, Melchiore Giganti, Gianluca Campo

**Affiliations:** 1 Cardiovascular Institute, Azienda Ospedaliero-Universitaria di Ferrara, 44124 Cona, Italy; 2 Department of Morphology, Surgery and Experimental Medicine, Section of Radiology, University of Ferrara, 44121 Ferrara, Italy

**Keywords:** coronary CTA, epicardial fat, plaque features, late iodine enhancement

## Abstract

Nowadays, coronary computed tomography angiography (CCTA) has a role of paramount importance in the diagnostic algorithm of ischemic heart disease (IHD), both in stable coronary artery disease (CAD) and acute chest pain. Alongside the quantification of obstructive coronary artery disease, the recent technologic developments in CCTA provide additional relevant information that can be considered as “novel markers” for risk stratification in different settings, including ischemic heart disease, atrial fibrillation, and myocardial inflammation. These markers include: (i) epicardial adipose tissue (EAT), associated with plaque development and the occurrence of arrhythmias; (ii) late iodine enhancement (LIE), which allows the identification of myocardial fibrosis; and (iii) plaque characterization, which provides data about plaque vulnerability. In the precision medicine era, these emerging markers should be integrated into CCTA evaluation to allow for the bespoke interventional and pharmacological management of each patient.

## 1. Introduction

Coronary computed tomography angiography (CCTA) is of paramount importance with regard to ischemic heart disease (IHD). CCTA is considered one of the best non-invasive imaging techniques for giving answers about coronary artery disease (CAD). The current guidelines about chronic coronary syndrome recommend CCTA as a first-line anatomical test for patients with a low and intermediate risk of CAD [1]. This technique plays a key role also in the treatment of acute chest pain with the aim of a better rule-in and rule-out of patients in the emergency department [2]. Additionally, CCTA is able to give other relevant information that can be considered “novel” markers for risk stratification in primary and secondary prevention. Data about epicardial adipose tissue (EAT) can help identify patients at higher risk of plaque development. The assessment of late iodine enhancement (LIE) can help identify scarred myocardium, for example, in patients with unknown previous myocardial damage. Finally, plaque characterization represents a new frontier in atherosclerosis evaluation and the identification of high-risk plaques without significant stenosis. Table 1 shows the technical aspects and the clinical meaning of these markers. The present review aims to summarize the main evidence about these three CCTA markers in order to highlight the importance of CCTA in the precision medicine era. 

## 2. Materials and Methods

Thanks to new technological developments, CCTA gives additional information in addition to the estimation of coronary stenosis. Three markers recently emerged as novel parameters in terms of diagnostic and prognostic CCTA performance: EAT quantification, LIE, and plaque characterization assessment. Each marker was demonstrated to predict adverse events and its identification by CCTA allowed a better risk stratification and management of patients [3,4,5]. 

A Medline search of full-text articles published in English until February 2020 was performed.

Overall, 679 records for EAT, 117 records for LIE, 903 records for plaque characterization were identified. For each parameter, the search terms were as follows: ((coronary CTA) OR (cardiac computed tomography) OR (CTA) OR (coronary CT)) AND ((epicardial fat) OR (EAT) OR (epicardial adipose tissue)); ((coronary CTA) OR (cardiac computed tomography) OR (CTA) OR (coronary CT)) AND ((LIE) OR (late iodine enhancement)); ((coronary CTA) OR (cardiac computed tomography) OR (CTA) OR (coronary CT)) AND ((plaque composition) OR (plaque characterization)). Only papers published in English and in peer reviewed journals were selected. 

The quality of the selected papers was tested using MINORS criteria [6]. Unblinded reviewers quality assessment. Discrepancies between reviewers were resolved by consensus. The maximum score obtained was 14 and the minimum 8. In the present review, we only included the studies obtaining a score of 10. Therefore, a total of 86 papers were considered for this overview (EAT = 26; LIE = 22; plaque characterization = 32).

EPICARDIAL FAT

What is it?

EAT represents the fat layer around the heart, between the myocardium and the visceral layer of the pericardium. It is of particular interest because of its unique anatomic and physiologic relationship to the heart [3]. 

The EAT has several known physiological functions, including the following: protecting the coronary arteries from the mechanical stress of arterial pulse and cardiac contraction; controlling vascular tension; stimulating nitric oxide production; reducing oxidative stress; and managing thermogenic function against hypothermia. It is also metabolically active (secreting mediators and pro- and anti-inflammatory cytokines), functioning as a paracrine and endocrine organ in lipid and glucose homeostasis. Thanks to its ability to use free fatty acid (FFA), EAT can protect the myocardium from their cardiotoxic effect. Additionally, it produces adiponectin that protects coronary circulation, improves endothelial function, reduces oxidative stress, and indirectly decreases the level of interleukin-6 (IL-6) and C-reactive protein (CRP) [7,8]. 

**Figure 1:**
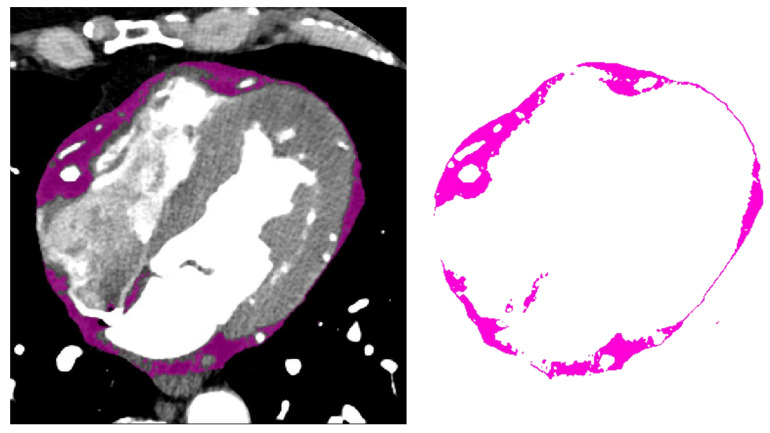
Epicardial fat assessment: purple contours show EAT volume with cardiac CT.

CCTA is the best technique for EAT assessment because of its high spatial resolution (Figure 1). EAT in non-contrast CCTA is visualized as a tissue with attenuation ranging from −190 to −30 Hounsfield Units around the heart. Volumetric assessment of EAT can be performed semi-automatically or by automated algorithms (quantifying both attenuation and volume of EAT) [9,10,11].

One of the main advantages of CCTA is the ability to assess the coronary tree and EAT simultaneously, providing information about the relationship between coronary atherosclerosis and fat adjacent to the vessel. Antonopoulos et al. assessed EAT attenuation developing the fat attenuation index (FAI), which was defined as the average EAT attenuation within a radial distance from the outer wall of the coronary artery equal to the mean diameter of the vessel. This parameter allows for the assessment of the size and lipid content of adipocytes in the proximity of the coronary arteries [12].

In recent years, researchers have established a relationship between EAT and heart diseases, including CAD, heart failure with preserved ejection fraction (HFrEF), and atrial fibrillation (AF) [13,14].

When and why to use it?

- Coronary Artery Disease

Inflammation can be involved in all stages of CAD, from initial atherogenesis to the progression of atherosclerotic lesions, and finally to plaque rupture and atherothrombosis. In this context, an EAT dysfunction seems to play a key role through the increased production of proinflammatory adipokines, failure of triglyceride storage, increased lipolysis, and release of free fatty acids [15]. 

Alexopoulos et al. demonstrated that the EAT volume was greater in the presence of CAD-obstructive noncalcified plaques, showing the association between EAT and the vulnerable plaques [16]. In a study combining a CT scan and intravascular ultrasound imaging (IVUS), Yamashita et al. demonstrated that EAT was associated with total coronary plaque burden and an increased vulnerability of the plaque (particularly in the right coronary artery and left anterior descending artery) [17]. These results confirmed that the amount of EAT and/or EAT proinflammatory state correlate with the severity of CAD and plaque vulnerability.

In view of its link to atherosclerotic plaque, several studies demonstrated that EAT was an independent predictor of major cardiovascular events (MACE). In particular, Mancio et al. conducted a metanalysis demonstrating the independent association between EAT volume and coronary artery stenosis, myocardial ischemia, and MACE [18]. Mahabadi et al. pointed out that EAT was associated with fatal and nonfatal coronary events in the general population and added information from cardiac CT above the Calcium score [19]. Goeller M et al. analyzed 456 asymptomatic subjects and showed that EAT density was significantly related to MACE (*p* = 0.029) [20]. Another study by Mahabadi et al. about secondary prevention found that patients with myocardial infarction have high EAT volume and attenuation [21].

In the CRISP-CT (Cardiovascular RISk Prediction using Computed Tomography) study, perivascular fat attenuation was assessed by developing the Fat Attenuation Index (FAI). The study demonstrated that FAI predicted all-cause and cardiac mortality over clinical risk factors [22]. Recently, a post hoc analysis of the CRISP-CT study reported that FAI provides incremental prognostic value over and above the presence of high-risk plaques on CCTA. Furthermore, fat attenuation could be useful for clinical risk assessment and also guide the deployment of targeted anti-inflammatory therapies in patients with stable CAD and residual inflammatory risk [23]. Table 2 offers a possible management algorithm for patients with a large amount of EAT.

- Atrial Fibrillation

EAT has emerged as a risk factor and independent predictor of AF development and recurrence after ablation [24]. The literature indicates several potential mechanisms linking EAT with AF, including the following: proinflammatory status of EAT; reactive oxygen species (ROS) released by EAT; fatty infiltration of the atrium; dysfunction of the autonomic nervous system. It has been postulated that the EAT can change electrophysiological characteristics and ionic currents via cytokines, adipokines, and adipocyte infiltration, causing the formation of the electrical substrate for AF. Other less understood potential mechanisms may explain the involvement of EAT in the pathogenesis of AF, such as the positive correlation between the total aromatase content of EAT and the occurrence/duration of triggered atrial arrhythmias [25,26].

In the Framingham Heart Study cohort, fat volume was an independent predictor of AF even after adjusting for other risk factors. In two recent meta-analyses, the association between AF and EAT was confirmed, and it was stronger with persistent AF than paroxysmal AF [27]. Other studies compared some well-established structural heart abnormalities linked to AF, such as left atrial size, with peri-atrial inflammation, as measured by adipose tissue attenuation on the CCTA. They found that the latter is strongly associated with AF regardless of the LA size. From a clinical point of view, it was shown that patients with a higher EAT volume had a worse outcome and earlier AF recurrences after transcatheter ablation [20,21,22,23,24,25,26,27,28,29,30].

The recent interest in therapies capable of reducing the volume of EAT and its link to the development of AF and AF recurrence after transcatheter ablation may open the way to new approaches for the treatment and prevention of this arrhythmia (Table 2).

- Heart Failure

EAT has been suggested to have a role in heart failure, particularly in patients with HFpEF. When fat cells become dysfunctional, they begin to produce proinflammatory factors that lead to chronic systemic inflammation. On one hand, through these inflammatory mechanisms mediated primarily by adipokines, “epicardial” obesity could cause adverse myocardial remodeling in HF, particularly in those with left ventricular ejection fraction >40%. On the other hand, epicardial fat may also negatively impact cardiac performance due to a direct mechanical effect caused by an increased pericardial restraint and enhanced ventricular interdependence [31,32].

The association between EAT thickness or volume and heart failure with reduced ejection fraction (HFrEF) is controversial. EAT parameters appeared either increased or, more frequently, decreased in HFrEF patients compared with healthy individuals. This variability may be explained by the presence of comorbidities, such as CAD, obesity, and diabetes, which may influence EAT volume in HFrEF. In addition, changes in metabolic and hemodynamic status that characterize HFrEF may modulate EAT volume. Critically ill patients with HFrEF may present a widespread systematic fat loss and, as a result, a reduced EAT volume [33,34]. 

Recent studies evaluated the response to CRT in patients with HFrEF. EAT thickness of the left atrioventricular groove was associated with total perfusion deficit of the left ventricle and left ventricular systolic dyssynchrony in patients with non-ischemic systolic HF. The EAT thickness of the AV groove had a predictive value for CRT response in patients with non-ischemic systolic HF [35]. 

LATE IODINE ENHANCEMENT

What is it?

In terms of tissue characterization, CCTA shares similar properties with cardiac magnetic resonance (CMR) in terms of tissue characterization. The iodine-based contrast media used in CCTA share some characteristics with gadolinium, which is used in CMR. In fact, the former show a delayed washout in scarred myocardium compared to the normal one, generating areas of late iodine enhancement (LIE) with a 5–15 min delayed CCTA scan [36,37]. The acquisition protocol for LIE-CT is currently based on two pillars. First, the administration of larger amounts of iodinated contrast medium (at least 1.5 mL/kg) when compared to the dose needed for coronary anatomy evaluation. Second, the acquisition of CT images with ECG gating after 8–10 min postcontrast administration. The continuous advancement in CT technology, in terms of increased spatial and temporal resolution, has led to a broader clinical application. This, combined with improvements in reconstruction algorithm and detector technology, has allowed for significant noise reduction in images acquired at low energy, expanding the application of CT to myocardial scar characterization [36].

In contrast to CMR, besides its suitability for coronary artery imaging, CCTA is widely used because of its shorter acquisition times, its accessibility, and its desirability in patients wearing a cardiac implantable device or undergoing dialysis. Moreover, LIE-CT, through a combined evaluation of myocardial scar and coronary arteries patency, potentially allows for the simultaneous detection of the culprit lesion and its related myocardial viability [36,37].

One of the most promising advances in this field is the use of dual-energy CT, which enables tissue characterization with extracellular volume (ECV) estimation and is considered a myocardial fibrosis equivalent when evaluated with CMR. Moreover, a strong correlation was seen between CMR-ECV and CT-ECV in assessing myocardial tissue in heart failure patients [4].

However, the routine application of cardiac CT and LIE is currently limited, mainly due to the lack of data confirming its diagnostic value. According to small studies, the inter-observer agreement between LIE-CT and LGE-CMR is dependent on the reader’s experience, with a per-patient overall accuracy of 95% and 88% for the most and the least experienced operator, respectively. In both per-segment and per-patient analyses, the specificity and the positive predictive value were excellent, regardless of the reader’s experience [38,39]. 

**Figure 2:**
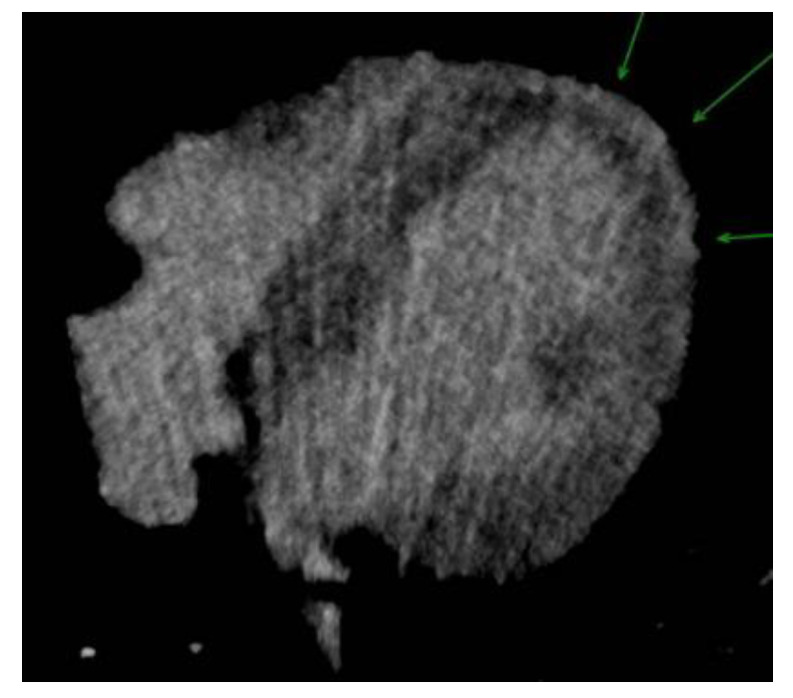
Late Iodine Enhancement: LIE in myocarditis.

The aim of the current section of this paper is to review the emerging application of cardiac CT, with specific reference to the LIE-CT role in detecting cardiac diseases and its implications in patient management (Table 2) (Figure 2).

When and why to use it?

- Coronary artery disease and heart failure

Aside from myocardial function evaluation, myocardial fibrosis detection has a significant prognostic value in ischemic heart disease.

Among the first reports of myocardial fibrosis by CT, there is a small autoptic study in 17 animals in which an accurate myocardial tissue characterization was demonstrated [40]. In 2005, the first-in-human study, comprising 28 patients with a history of myocardial infarction, showed an excellent agreement between CMR and CT in assessing fibrosis [41]

Three years later, an overall good agreement between CCTA and CMR plus invasive coronary angiography was demonstrated in the assessment of myocardial fibrosis and coronary anatomy in 71 patients with new onset ventricular disfunction [42].

Heart failure (HF) also provides a basis for the wide use of cardiac imaging. According to the current European Society of Cardiology (ESC) guidelines, CT plays a role in ruling out coronary artery stenosis in patients with a low-to-intermediate pre-test probability of CAD, and those with equivocal non-invasive stress tests (class of recommendation IIa) [43]. CAD is recognized as the main cause of HF in about 50% of the cases; thus, the appropriate evaluation of both coronary arteries and myocardial fibrosis is necessary in this category of patients [44]. Small studies have shown that cardiac CT is a feasible, safe, and effective imaging tool in determining the underlying etiology of newly diagnosed HFrEF, as it allows coronary arteries and myocardial fibrosis to be examined simultaneously [40].

As with LGE-CMR, the infarcted area can be detected by LIE-CT via hyper-enhancement. Previous studies highlighted that the presence of both hyper-enhancement and hypo-enhancement showed a better correlation with microvascular obstruction, wall thinning, cardiac remodeling, and ejection fraction compared to the presence of hyperenhancement alone and was predictive of future major adverse cardiovascular events (MACE) [45,46].

Moreover, in patients with heart failure, LIE-CT imaging has shown a good agreement with CMR in the localization and pattern recognition of myocardial fibrosis [47,48].

- Cardiomyopathies and myocarditis

Previous studies investigated LIE-CT in different clinical scenarios, such as hypertrophic cardiomyopathy (HCM) and cardiac sarcoidosis, showing a good sensitivity in detecting myocardial scars [49,50].

It is worth noting that the AHA/ACC 2020 guidelines on the diagnosis and treatment of HCM included cardiac CT as an alternative technique to CMR for myocardial tissue characterization and scar detection when considering ICD implantation for primary prevention [51]. At the same time, it is of paramount importance to recognize that even CCTA images acquired for coronary anatomy evaluation can give information about the myocardial thickness and the myocardial fat infiltration, and it is mandatory to observe and report these non-coronary but cardiac findings [52,53,54].

Among the possible clinical applications of CCTA, one of the most promising ones is the evaluation of anatomical substrate in patients with ventricular arrhythmias scheduled for transcatheter ablation. CCTA with LIE enables concomitant myocardial tissue characterization and coronary anatomy evaluation; moreover, it can allow accurate pre-procedural planning in case of an epicardial approach [55].

Few emerging data showed the usefulness of LIE in myocarditis. Bouleti C et al. performed a proof-of-concept study demonstrating that spectral CT was a valid alternative to CMR for the detection and assessment of myocardial inflammation in acute myocarditis. Considering that myocarditis can be misclassified as ACS, CT could play a key role in ruling out ACS and highlighting inflammation by LIE. This concept gained paramount importance during the SARS-CoV-2 pandemic. By using dedicated LIE, CCTA was useful in the diagnosis of COVID-19-related myocarditis [56]. For the aforementioned reasons, CCT with LIE may be considered when CMR is contraindicated or unfeasible as it is proving to be an excellent alternative tool in different clinical scenarios. 

PLAQUE CHARACTERIZATION

What is it?

CCTA can inform us about these high-risk characteristics and allows us to identify the “vulnerable” plaque. Histopathological studies first investigated the characteristics of a vulnerable plaque, which included the following: necrotic core, thin fibrous cap, spotty calcification, positive remodeling, and inflammation involving plaque and perivascular tissues [57,58].

**Figure 3:**
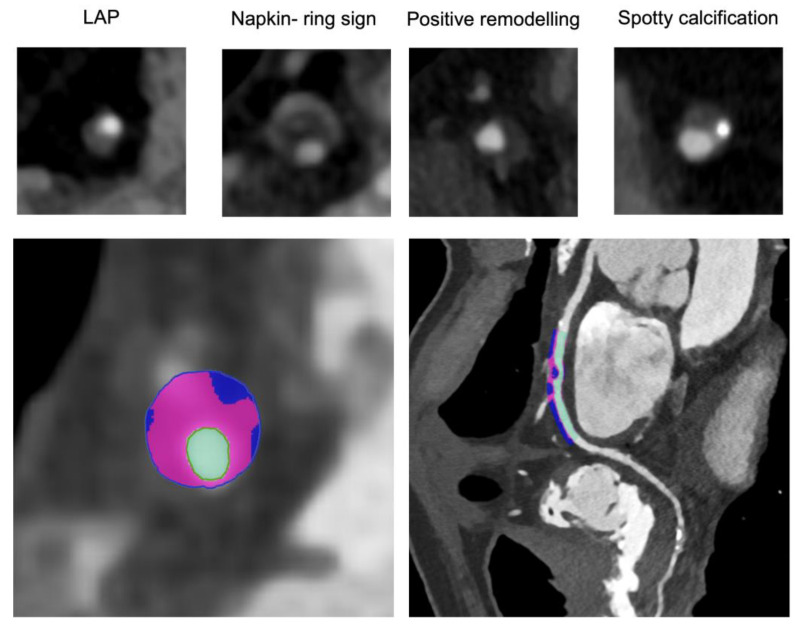
Plaque characterization: the high-risk plaque features and an example of plaque composition analysis. LAP = low attenuation plaque.

CT vascular tissue radiodensity has a good correlation with histological composition, with calcified plaques corresponding to higher attenuation values (e.g., >465 Hounsfield Units HU), compared with fibrotic (e.g., 65–260 HU) and low-attenuation lesions with a necrotic core (e.g., −1 to 64 HU) [59]. So, with CCTA, we can also obtain qualitative information (i.e., low-attenuation plaque, napkin-ring sign, and spotty calcification) and not just quantitative information (i.e., total/calcified/noncalcified plaque burden, diameter stenosis, remodeling index) [60]. High-risk plaque features were described as follows [3,61,62,63,64] (Figure 3):

- Low attenuation plaque (LAP) is traditionally defined as a plaque area with a mean attenuation of <30 HU and reflects a lipid-rich necrotic core, an extracellular conglomerate within the intima induced by the necrosis and apoptosis of lipid-laden macrophage foam cells.
- A napkin-ring sign is defined as a low attenuation core, with a thin hyper-attenuated ring around a necrotic core.
- Positive remodeling is a relative increase in the cross-sectional diameter of a lesion compared with a proximal, reference segment of 1.1 or higher. Positive remodeling is related to compensatory mechanisms of coronary autoregulation, which maintain a stable vessel area even when the plaques extend more than 40% of the total lumen. Vascular remodeling can then be detected on CCTA as a relative increase in vascular diameter around the plaque.
- Spotty calcification is defined as a small (<3 mm), dense (>130 HU) plaque component surrounded by noncalcified plaque tissue.

When and why to use it?

Although the obstructive/non-obstructive dichotomy still guides main clinical decisions today, CCTA can give us more information about plaques [65].

As a matter of fact, a patient with CAD is referred to invasive coronary angiography (ICA) depending on the percentage of stenosis, as it is the most clinically validated element to guide revascularization [66].

Despite this, it is widely demonstrated that the majority of culprit lesions in ACS arise from non-obstructive plaques with high-risk characteristics—in the ICONIC trial, 31% of culprit lesions in ACS had high-risk characteristics, and 52% of non-ACS patients with high-risk plaque features experienced an ACS during the follow-up. For this reason, improving cardiovascular risk prediction requires a more comprehensive, individualized assessment of coronary atherosclerosis and patient-specific vascular biology [67]. So, a plaque characterization should be performed in all patients undergoing CCTA in order to achieve the best treatment strategy (Table 2).

Narula et al. defined the fibrous cap thickness as the best predictor of vulnerable plaques [68]. CT lacks the spatial resolution to detect thin cap fibroatheroma, but CT-derived high-risk plaque features can discriminate thin cap fibroatheroma lesions from non-thin cap fibroatheroma lesions [69]. In this context, Otsuka K et al. analyzed 895 patients who underwent CCTA and who were followed for more than one year. The study demonstrated that both LAP (*p* = 0.007) and napkin ring sign (*p* > 0.001), especially the latter, were independent predictors of future ACS [70].

Several studies focused on the relationship between positive remodeling and LAP and prognosis.

In the NXT study (Analysis of Coronary Blood Flow Using CT Angiography: Next Steps), this plaque-level high-risk feature was associated with an increased presence of ischemia, regardless of the severity of stenoses [71]. Despite a low degree of stenosis, the reason why low LAPs are more frequently associated with ischemia is due to the inflammation they are associated with; in fact, inflammation causes an imbalance between vasoconstricting and vasodilating substances [72].

In a large longitudinal study, Motoyama et al. reported that high-risk plaques with positive remodeling and/or LAP were associated with an increased risk of future ACS [73].

In a sub-analysis of the PROMISE trial, the presence of spotty calcification (i.e., as well as the presence of LAP, napkin ring sign, and positive remodeling) was associated with higher rates of major adverse cardiac events (death, MI, or hospitalization for unstable angina) at 25 months in 4415 patients with stable symptoms (HR: 2.73). Furthermore, in this trial, it was shown that the presence of vulnerable plaque features is associated with adverse events, even in the absence of obstructive coronary disease [74]. The presence of spotty calcifications has been shown to be more frequent in patients with ACS than in patients with stable CAD; on the contrary, large calcifications are more frequently associated with conditions of stability [75].

Additionally, in the SCOT-HEART study, which included patients with stable chest pain, the presence of at least one of the high-risk characteristics was shown to be associated with a higher risk of myocardial infarction and cardiovascular death at 5 years [76]. 

Given the large amount of strong evidence, the Coronary Artery Disease Reporting and Data System (CAD-RADS) guidelines recommend medical professionals to report the presence of plaque vulnerability if at least two of the aforementioned high-risk features are present in the CCTA study [77].

Furthermore, with the advent of increasingly sophisticated plaque characterization software, it is possible to precisely quantify the plaque volume and assess the burden of different plaque types, including calcific, non-calcific, and low attenuation plaque. It has recently been shown that the use of 256-slice CTA to quantify the plaque volume has an excellent correlation with IVUS [78].

The total atheroma volume in the non-obstructive lesions is a factor that is associated with plaque progression to obstructive lesions [79]. In the CAPIRE (Coronary Atherosclerosis in Outlier Subjects: Protective and Individual Risk Factor Evaluation) study, the plaque volume and, in particular, the non-calcified plaque volume was an important predictor of cardiovascular events. In fact, it was a stronger predictor of adverse events when compared to lumen stenosis and clinical risk [80]. 

Finally, in the SCOT-HEART study, the factor most associated with the risk of myocardial infarction was the low attenuation plaque burden. This was a better predictor than coronary stenosis severity, coronary Calcium Score, cardiovascular risk score, and also atheroma volume [81]. 

Conte et al., in a huge prospective study with a follow-up of 98 months, evaluated plaque burden and other plaque features related to ACS and cardiovascular mortality in patients without obstructive coronary disease. This study showed that positive remodeling, LAP, a plaque burden of more than 0.7, or napkin ring sign are the most important predictors of death or ACS [82]. 

The presence of high-risk characteristics has a different prognostic implication according to FFR value. The 3V FFR-FRIENDS study highlighted that the presence of more than three high-risk plaque characteristics is associated with adverse cardiac events, particularly in deferred lesions with a FFR greater than 0.8 [83].

Recently, several authors have tried to make up a risk score in order to use all CT information to improve the risk stratification of each patient. Two risk scores were created. The first, the Leaman CT score (CT-LeSc), combines the type of plaque (calcified or non-calcified), the degree of stenosis, and the coronary plaque location. In the validation study of this score, it was shown that patients with a CT-LeSc greater than five with non-obstructive CAD had an adverse event-free survival comparable to patients with obstructive CAD [84,85]. This score was also validated in the CONFIRM study, wherein 2402 patients without prior CAD history who underwent CCTA that showed non-obstructive CAD were enrolled. A complete analysis of plaque composition was performed. CT-LeSc was an independent predictor of MACE, improving the prognostic stratification of patients with non-obstructive CAD [86]. 

The second risk score is the LEIDEN CT risk score, which assessed the degree of coronary artery stenosis (>50% or <50%), the type of plaque (calcified, non-calcified, or mixed), and the plaque location. A higher Leiden CTA score was associated with a 5-year all-cause mortality or MI [87]. 

Finally, in the setting of plaque analysis, CCTA is also used to evaluate changes in atherosclerotic plaque following pharmacological and non-pharmacological therapies. In the PARADIGM (Progression of atherosclerotic plaque determined by Computed Tomographic Angiography Imaging) study, statin therapy reduced the high-risk characteristics of plaque. In particular, this therapy converted vulnerable plaques into calcified plaques, reduced the progression of calcified plaques into high-risk plaques, and reduced plaque burden [88]. 

In the EVAPORATE (Effect of Vascepa on Improving Coronary Atherosclerosis in People With High Triglycerides Taking Statin Therapy) trial, therapy with statins and icosapent ethyl resulted in a reduction in LAP volume, compared to therapy with statin alone [89]. 

The role of lifestyle intervention was also recently evaluated. In a study of 92 patients, a healthy lifestyle (healthy diet and physical activity) associated with optimized medical therapy (OMT) was shown to reduce high-risk plaque and calcified plaque progression compared to OMT alone [90]. 

## 3. Future Perspectives

In recent years, new technologies improved the performance of CCTA. New generation CTs are characterized by the option of a whole heart coverage, higher spatial and temporal resolution, and faster scan mode than prior-generation scan [3]. These features improved the quality of images. Recent advances in technology focused on a reduction in contrast media dose and radiation exposure. On one hand, the latest CT scanners allow a short acquisition time, requiring a reduced contrast media volume for coronary opacification. On the other hand, in order to achieve a reduction in radiation exposure, some tools are now emerging, such as true cardiac-capable photon counting detectors with greater spatial resolution [91]. These advances also have the ability to perform an in-deep plaque analysis [74].

Future perspectives have to take into account machine learning and deep learning, which represent promising tools in CCTA analysis. The CLARIFY study analyzed artificial intelligence in coronary artery segmentation in 232 patients undergoing CCTA, demonstrating that the performance of artificial intelligence was excellent for the accuracy, sensitivity, specificity, positive predictive value, and negative predictive value [92]. These tools could significantly overcome the limitations related to human interpretation.

## 4. Conclusions

Today, CCTA represents a front-line approach for the assessment of CAD. Furthermore, CCTA allows for the early identification of atherosclerosis and high-risk plaque features, recognition of EAT abnormalities, and diagnosis of unknown ischemic and non-ischemic heart disease thanks to LIE. As reported in the present review, all these data play a key role in the prognostic assessment of patients undergoing CCTA. Emerging CCTA risk scores have been provided in order to reinforce the link between imaging characteristics and clinical aspects. Future perspectives should move in this direction. The aim of CCTA analysis should be to help the management strategy of each patient beyond the mere quantification of coronary stenosis. Imaging data should be integrated with laboratory and clinical information in order to choose the best-tailored therapeutic option for each patient. 

## Figures and Tables

**Table 1:** Technical aspects and clinical meaning of each CT marker.

Parameters	Technical Aspects	Clinical Meaning
EAT	No need of contrast media Slice thickness 2.5–3 mm Ad hoc software for volume calculation	Help in identification of patients at risk of vulnerable plaque development
LIE	CCTA standard protocol Acquisition of images at 6–8 min after contrast injection	Help in diagnosis of HF etiology, rule-in and rule-out in myocarditis
High-risk plaque	CCTA standard protocol Availability of plaque reconstruction software	Help in identification of patients with a high risk of adverse events

**Table 2:** Possible clinical implication algorithm based on CT markers.

Clinical Scenario	Possible Clinical Implication
Large EAT volume in dysmetabolic patient	Optimization of lipid-lowering therapy, closer cardiovascular risk factors monitoring
Large EAT volume in CAD patient	Optimization of lipid-lowering therapy
Large EAT volume in AF patient	Pharmacological rate/rhythm control if possible
Large EAT volume in HF	Consider EAT with other validated parameters for CRT response
LIE related to unknown previous myocardial infarction	CMR for estimation of viability, coronary angiography *
LIE related to acute myocarditis	Medical therapy and CMR follow up at 3–6 months *
LIE related to HCM	Closer follow up for arrhythmogenic risk *
High-risk plaque features with significant stenosis	Coronary angiography
High risk plaque features without stenosis	Optimization of lipid-lowering therapy
High-risk plaque features with intermediate stenosis	Optimization of lipid-lowering therapy, antiplatelet therapy, stress test

* Further data are needed.

## Data Availability

Not applicable.
